# Genetic dissection of eating and cooking qualities in different subpopulations of cultivated rice (*Oryza sativa* L.) through association mapping

**DOI:** 10.1186/s12863-020-00922-7

**Published:** 2020-10-14

**Authors:** Chunfang Zhao, Ling Zhao, Qingyong Zhao, Tao Chen, Shu Yao, Zhen Zhu, Lihui Zhou, Altafhusian B. Nadaf, Wenhua Liang, Kai Lu, Yadong Zhang, Cailin Wang

**Affiliations:** 1 Institute of Food Crops, Jiangsu Academy of Agricultural Sciences, Jiangsu Rice Engineering Research Center, National Center for Rice Improvement (Nanjing), Nanjing, 210014 China; 2 Department of Botany, Savitribai Phule Pune University, Pune, 411007 India

**Keywords:** Rice (*Oryza sativa* L.), Eating and cooking quality, Population structure, Subpopulation, Association mapping

## Abstract

**Background:**

Eating and cooking qualities (ECQs) of rice (*Oryza sativa* L.) determine consumer acceptance and the economic value of rice varieties. The starch physicochemical properties, i.e. amylose content, gel consistency, gelatinization temperature and pasting viscosity are important indices for evaluating rice ECQs. Genetic factors are required for development of rice varieties with excellent ECQs and association mapping is one of the promising approaches for discovering such associated genetic factors.

**Results:**

A genome-wide association mapping was performed on a set of 253 non-glutinous rice accessions consisting of 83 *indica* and 170 *japonica* cultivated rice varieties through phenotyping for 11 ECQ traits in two consecutive years and genotyping with 210 polymorphic SSR and candidate-gene markers. These markers amplified 747 alleles with an average of 3.57 alleles per locus. The structure, phylogenetic relationship, and principal component analysis indicated a strong population differentiation between *indica* and *japonica* accessions and association mapping was thus undertaken within *indica* and *japonica* subpopulations. All traits showed a large phenotypic variation and highly significant phenotypic correlations were present between most of traits. A total of 33 and 30 loci were located for 11 ECQs in *indica* and *japonica* subpopulations respectively. Most of associated loci were overlapped with starch synthesis-related genes (SSRGs), and the *Wx* locus gathered 14 associated loci with the largest effects on amylose content, gel consistency and pasting viscosities. Eight subpopulation specific markers, *RM588*, *Wx-(CT)*_*n*_, *SSI* and *SBE1* for *indica* subpopulation and *RM550*, *Wx*^*mp*^, *SSIIa* and *SBE4* for *japonica* subpopulation, were identified, suggesting alleles of SSRGs showed the subspecific tendency. Nevertheless, allelic variation in *SSIIa* showed no tendency towards subspecies. One associated maker *RM550* detected in *japonica* subpopulation for amylose content and pasting viscosity was verified a potential novel and stably expressed locus and could be selected for further fine mapping.

**Conclusion:**

This study illustrated the potential for dissecting genetic factors of complex traits in domesticated rice subspecies and provided highly associated markers to facilitate marker-assisted selection for breeding high-quality *indica* or *japonica* rice varieties.

## Background

Rice (*Oryza sativa* L.) is the most important food crop in the world and feeds more than half of the world’s population. Grain physicochemical characteristics of the milled rice play critical roles in determining eating and cooking qualities (ECQs) of rice. Amylose content (AC), Gel consistency (GC), Gelatinization temperature (GT) and pasting properties are widely recognized as the important indices in determining ECQs [1].

The genetic basis of ECQs have been extensively studied in rice. Amylose content is the most important chemical property affecting ECQs, mainly controlled by the *Wx* gene encoding granule-bound starch synthase I (GBSSI), responsible for amylose synthesis in endosperm. *Wx*^*a*^ and *Wx*^*b*^ are the most common functional alleles for AC regulation in non-glutinous rice [2, 3]. In comparison with *Wx*^*a*^, *Wx*^*b*^ has G-T mutation at the splicing site of the first intron that reduces the splicing efficiency of precursor mRNA and the amount of GBSSI, thus leading to the reduction of AC in the target material. Moreover, single nucleotide polymorphisms (SNPs) in exons 4 (*Wx*^*op*^, *Wx*^*mq*^ and *Wx*^*mp*^) and 6 (*Wx*^*in*^) cause amino acid substitutions, resulting in lower AC values and pasting viscosity than their background materials [4–6]. In addition, microsatellite variations of (CT)_n_ in noncoding region of the *Wx* gene was found to be in significant correlation with AC [7]. Further, the findings of *dull* mutants uncovered the complex genetic regulation of amylose synthesis [8, 9]. GC is used as an indicator to measure cold paste viscosity of rice flour, especially reflecting the textural property in cooked rice [10]. GC is highly negatively associated with AC, i.e., a rice variety with high AC tends to show low GC or hard gel after cooling. Map-based cloning of the *qGC-6* locus revealed that *Wx* is the major gene responsible for GC [11], and the same conclusion was drawn through association and linkage mapping [12, 13]. Rapid Visco Analyzer (RVA) plays an essential role in estimating ECQs and processing qualities of rice, which can distinguish eating quality of rice varieties with similar AC by the variation trend of pasting viscosity profiles. RVA parameters are mainly controlled by the *Wx* gene, but several minor-effect genes or QTL other than *Wx* were found to be associated with RVA parameters [14, 15]. GT, indicated by the alkali spreading value (ASV), predicts rice cooking quality. The gene *ALK*, which is also known as *SSIIa*, has been reported to regulate rice GT [16]. The detection of QTL and association loci indicted that *SSIIa* has major effects on GT, and minor effects on GC and/or most of RVA parameters [13, 14]. Two functional SNPs in exon 8 of *SSIIa*, GC/TT and G/A, are responsible for SSIIa activity and lead to different GT of rice flour and chain length distribution of amylopectin [1]. The GC/TT polymorphism can differentiate rice varieties with high and intermediate GT from low GT rice varieties. The G/A SNP is crucial for SSIIa activity; the allele A causes SSIIa enzyme inactive regardless of GC/TT polymorphism, though A allele is rare in natural rice population [1, 17]. Besides *Wx* and *SSIIa*, the genetic effects of other starch synthesis related genes (SSRGs) such as *SSI*, *PUL*, *SSIIIa*, *SBE1* and *SBE3* on rice ECQs have been widely reported [18–20]. Additionally, dozens of QTL for each trait of ECQs located on all of rice chromosomes using bi-parental populations could be retrieved in the Q-TARO QTL database and the Gramene QTL database. However, the traditional bi-parental population has showed several limitations, especially in finding the minor QTL. The genetic basis of the variation in ECQs is not yet fully understood among the non-glutinous rice varieties.

Association mapping is a powerful method to correlate genetic loci and phenotypic performance based on a natural population. Association mapping, especially candidate-gene association mapping, has been commonly used to detect QTL for grain qualities in rice. Tian et al. [13] found that the starch synthesis-related genes cooperated with each other to form a regulatory network controlling ECQs. Yan et al. [19] tested 118 waxy rice varieties using 17 SSRGs with 43 genic markers and found that 10 SSRGs are involved in regulating the RVA parameters. However, candidate-gene association mapping may easily miss some unknown loci, while genome-wide association mapping is a more effective strategy to comprehensively identify causal genetic variations in the whole genome [21].

In the present study, we used genome-wide association mapping in combination with SSRGs candidate-gene markers to explore the loci for 11 ECQ traits in different subpopulations of *indica* and *japonica* rice varieties and compared the subspecies tendency of the association loci. The purpose is to detect specific QTL of ECQs within rice subspecies and to elucidate the relationships of SSRGs and ECQs based on the similar genetic background.

## Results

### Molecular marker polymorphism

Two hundred ten molecular markers involving 34 candidate-gene markers from 15 SSRGs, 19 sequence tagged site (STS) and 157 SSR markers detected a total of 747 alleles in 253 rice accessions. Numbers of alleles ranged from 2 (at locus Wx-S1_Chr.6) to 9 (at locus RM81_Chr.8) with an average of 3.57 alleles per locus. The genetic diversity averaged 0.453 ranging from 0.008 (at locus STS2-1_Chr.2) to 0.782 (at loci RM336_Chr.7 and RM1342_Chr.2) (Additional file 1: Table S1). The polymorphism information content (PIC) had a mean of 0.402 ranging from 0.008 (at locus STS2-1_Chr.2) to 0.753 (at locus RM1342_Chr.2) with a major distribution between 0.35 and 0.55 (Additional file 2: Fig. S1). Fifty-two (24.6%) and thirty-four (16.1%) markers were highly informative (PIC> 0.5) and slightly informative (PIC< 0.25), respectively. One hundred and twenty five markers (59.2%) were found moderately informative (0.5 > PIC> 0.25).

### Population structure

**Fig. 1:**
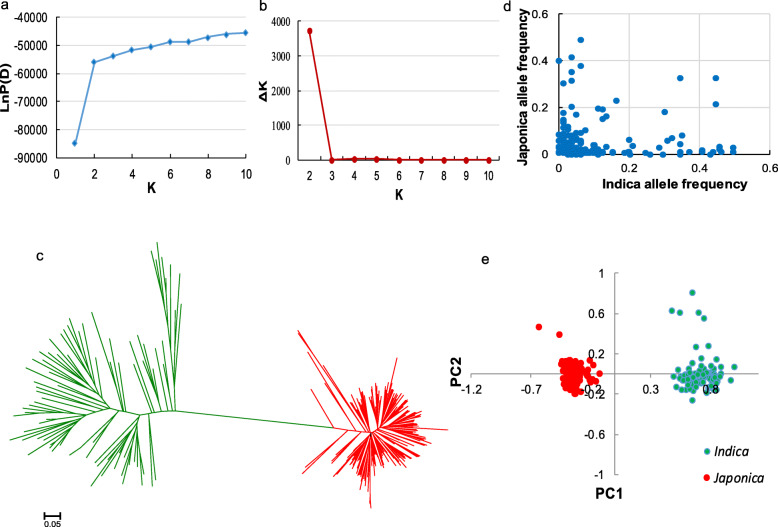
Population structure and divergence of 253 rice cultivars. **a** The mean LnP(D) changed with the number of subgroups. **b** The ΔK value changed with the number of subgroups. **c** Neighbor-joining tree constructed from simple matching distance of all markers. Red, *japonica*; green, *indica*. **d** Comparison of allele frequencies between *indica* and *japonica*. For each marker, we identified the minor allele across all landraces and then calculated the frequency of this allele in *indica* and *japonica*. **e** PCA plots of the first two components of 253 rice cultivars

The analysis of model-based population structure provided evidence for a significant population structure. The log-likelihood values increased with the increase in the model parameter K (Fig. 1a). ΔK, as the diagnostic criterion, was thus used to determine a suitable value for K. The highest ΔK value was obtained at K = 2 (Fig. 1b), indicating the whole population composed of 253 rice cultivars could be divided into two subpopulations. The neighbor-joining tree constructed based on the Nei’s genetic distances showed that the entire population could be clearly divided into two groups belonging to the *indica* and *japonica* rice accessions (Fig. 1c), that is in consistent with the results from the structure analysis. The *indica* subpopulation consisted of 81 *indica* accessions, while the *japonica* subpopulation included all of the 170 *japonica* accessions as well as 2 *indica* accessions from South Korea. Through screening minor allele frequency of all markers, dozens of markers were found highly fixed in one subpopulation (Fig. 1d), which may strongly affect the *indica*-*japonica* differentiation. Furthermore, PCA plots of the first two component clearly differentiated the whole population into two subpopulations (Fig. 1e), which corresponded with the results of population structure and neighbor-joining tree. The population-differentiation statistic (*F*_ST_) between the *indica* and *japonica* subpopulations was estimated at 0.75, indicating an extremely strong population differentiation.

**Fig. 2:**
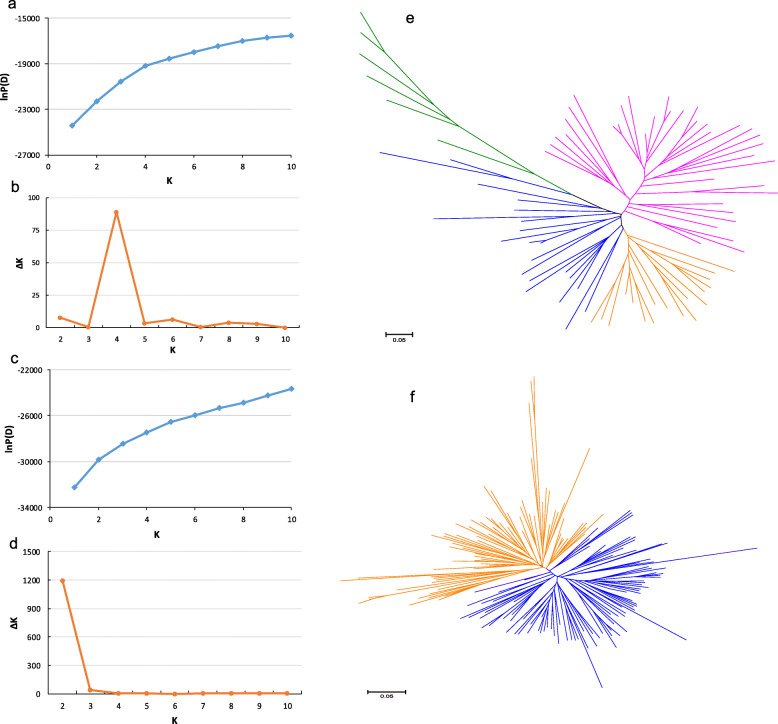
The analysis of population structure and phylogenetic relationship in *indica* and *japonica* subpopulations. **a-b** The mean LnP(D) (**a**) and ΔK value (**b**) calculated in the *indica* subpopulation. **c-d** The mean LnP(D) (**c**) and ΔK value (**d**) calculated in *japonica* subpopulation. **e-f** The Neighbor-joining tree constructed from 81 individuals in *indica* subpopulation (**e**) and 172 individuals in *japonica* subpopulation (**f**)

We further evaluated genetic structure in the two subpopulations. By K value analysis, the *indica* and *japonica* subpopulations could be divided into four and two subgroups, respectively (Fig. 2a-d). The neighbor-joining tree of *indica* and *japonica* subpopulations showed a consistent divergent subgroups with population structure analysis (Fig. 2e and f). Among the four subgroups of *indica*, *F*_ST_ was estimated at 0.215 suggesting a moderate level of differentiation within *indica* subpopulation. The genetic differentiation within *japonica* subpopulation was significantly less (*F*_ST_ = 0.057) than that in *indica* subpopulation, implying the fixation of alleles and inbreeding.

### Whole-genome patterns of linkage disequilibrium (LD)

LD analysis was performed for the *indica* and *japonica* subpopulations cultivars based on 210 markers (Additional file 2: Fig. S2) since a strong population differentiation was found in the whole set of rice accessions. In *indica* subpopulation, out of 21,310 pairs, 21,251 pairs (99.72%) showed LD with a D’ average of 0.36. The loci number with LD supported by D’ > 0.5 was 5204 and occupied 24.42%. In *japonica* subpopulation, 21,893 (99.90%) among 21,915 pairs showed LD, the loci with LD supported by D’ > 0.5 occupied 33.34% and the D’ average of LD pairs was 0.43. This indicated that LD level was higher in *japonica* subpopulation than in *indica* subpopulation, and the *japonica* subpopulation might have undergone a stronger manual selection than *indica* subpopulation.

### Phenotypic variations and correlations

The mean value, coefficient of variation (CV) and differences for 11 ECQ traits in the whole population and two subpopulations across 2 years are depicted in Table 1. Significant variations were observed in almost all of the investigated traits except for PeT and PT in either whole population or each subpopulation in both years. The largest coefficient of variation was found in SBV ranging from 259.6 to 814.5%. For the AC trait, the mean values were 17.3% (17.7%), 21.3% (22.7%) and 15.3% (15.3%) in the whole population, *indica* and *japonica* subpopulations in 2015 (2016), respectively. The mean values of GC were 70.6 (69.3), 58.2 (55.0) and 76.6 (76.3) in three panels in 2015 (2016), respectively. There was no significant difference for AC and GC between years, but the dramatic difference was found between two subpopulations. The mean values of ASV were 4.6 (3.2), 4.0 (2.8) and 4.9 (3.4) in three panels in 2015 (2016), respectively. The differences for ASV were significant between years and between subpopulations. The *indica* subpopulation had larger AC, and smaller GC and ASV in comparison with the *japonica* subpopulation. For the 8 parameters of RVA profile, the differences of most of parameters in three panels were significant between years, except for the PV, CV, SBV and Ptemp in *indica* subpopulation. There were also significant differences for most of RVA parameters except for PeT between subpopulations. Most of the RVA parameters had higher average value in *indica* subpopulation than in *japonica* subpopulation, while BDV and PeT showed the contrary values.

**Table 1:** Year wise comparative phenotypic performance in terms of eating and cooking qualities in the whole population and two subpopulations of cultivated rice

Trait	Year	Whole population			*indica* subpopulation			*japonica* subpopulation			Difference (%)
		Mean ± SD	Range	CV%	Mean ± SD	Range	CV%	Mean ± SD	Range	CV%	
AC (%)	2015	17.3 ± 4.4	8.0 ~ 28.5	25.4	21.3 ± 5.3	12.5 ~ 28.5	24.9	15.3 ± 1.93	8.0 ~ 19.0	12.6	28.1**
	2016	17.7 ± 5.3	8.0 ~ 30.9	29.9	22.7 ± 6.2	12.3 ~ 30.9	27.3	15.3 ± 2.2	8.0 ~ 19.8	14.4	32.7**
		ns			ns			ns			
GC (mm)	2015	70.6 ± 19.7	25.0 ~ 122.0	27.9	58.2 ± 25.4	25.0 ~ 122.0	43.6	76.6 ± 12.5	44.0 ~ 113.0	16.3	−31.7**
	2016	69.3 ± 21.0	25.5 ~ 103.5	30.3	55.0 ± 27.4	25.5 ~ 103.5	49.8	76.3 ± 11.9	48.0 ~ 99.5	15.6	−38.7**
		ns			ns			ns			
ASV (grade)	2015	4.6 ± 1.6	1.0 ~ 7.0	34.8	4.0 ± 1.8	1.0 ~ 7.0	45	4.9 ± 1.3	2.0 ~ 6.5	26.5	− 21.8**
	2016	3.2 ± 1.4	1.0 ~ 7.0	43.8	2.8 ± 1.4	1.1 ~ 7.0	50	3.4 ± 1.3	1.0 ~ 6.0	38.2	−21.3**
		**			**			**			
PV (RVU)	2015	219.6 ± 35.1	122.3 ~ 306.4	16.0	240.2 ± 38.0	122.3 ~ 306.4	15.8	209.6 ± 28.8	139.6 ~ 296.7	13.7	12.7**
	2016	230.2 ± 36.8	98.8 ~ 304.4	16.0	245.1 ± 41.0	98.8 ~ 304.4	16.7	222.9 ± 32.3	123.0 ~ 284.3	14.5	9.1**
		**			ns			**			
HPV (RVU)	2015	105.1 ± 32.5	47.7 ~ 214.0	30.9	130.7 ± 35.8	67.4 ~ 214.0	27.4	92.7 ± 21.7	47.7 ~ 157.6	23.4	29.1**
	2016	142.1 ± 38.4	39.3 ~ 246.3	27.0	157.7 ± 42.3	69.6 ~ 246.3	26.8	134.5 ± 33.9	39.3 ~ 212.5	25.2	14.7**
		**			**			**			
CPV (RVU)	2015	203.8 ± 61.6	71.4 ~ 415.5	30.2	261.5 ± 68.3	164.2 ~ 415.5	26.1	175.6 ± 31.1	71.4 ~ 244.2	17.7	32.8**
	2016	224.7 ± 52.0	77.7 ~ 354.9	23.1	259.3 ± 56.2	169.5 ~ 354.9	21.7	207.7 ± 40.2	77.7 ~ 296.8	19.4	19.9**
		**			ns			**			
BDV (RVU)	2015	114.5 ± 26.8	36.5 ~ 194.2	23.4	109.5 ± 37.0	36.5 ~ 194.2	33.8	117.0 ± 19.6	70.5 ~ 179.0	16.8	−6.9*
	2016	88.1 ± 31.0	26.5 ~ 177.0	35.2	87.4 ± 39.6	29.2 ~ 177.0	45.3	88.5 ± 26.0	26.5 ~ 162.4	29.4	−1.20
		**			**			**			
SBV (RVU)	2015	−15.9 ± 54.09	− 152.3 ~ 165.6	340.0	21.3 ± 72.8	− 120.3 ~ 165.6	341.8	−34.0 ± 28.0	−152.3 ~ 33.1	82.4	259.6**
	2016	−5.5 ± 44.8	− 125.4 ~ 95.08	814.5	14.2 ± 57.6	−107.5 ~ 95.1	405.6	−15.2 ± 33.2	− 125.4 ~ 51.3	218.4	206.8**
		*			ns			**			
CSV (RVU)	2015	98.7 ± 35.2	0.1 ~ 242.6	35.7	130.7 ± 41.1	73.8 ~ 242.6	31.4	83.0 ± 16.4	0.1 ~ 110.7	19.5	36.5**
	2016	82.6 ± 21.8	37.0 ~ 149.8	26.4	101.6 ± 23.4	64.0 ~ 149.8	23	73.3 ± 13.3	37.0 ~ 98.3	18.1	27.9**
		**			**			**			
PeT (min)	2015	5.9 ± 0.4	3.5 ~ 6.7	6.8	5.9 ± 0.5	3.5–6.7	8.5	5.9 ± 0.3	5.1 ~ 6.6	5.1	−0.4
	2016	6.2 ± 0.4	5.1 ~ 7.0	6.5	6.1 ± 0.3	5.4–6.8	4.9	6.2 ± 0.4	5.1 ~ 7.0	6.5	−1.10
		**			**			**			
Ptemp (°C)	2015	75.0 ± 4.1	68.1 ~ 88.6	5.5	78.0 ± 4.0	68.8 ~ 87.8	5.1	73.5 ± 3.2	68.1 ~ 88.6	4.4	5.8**
	2016	76.6 ± 3.9	68.8 ~ 90.2	5.1	78.7 ± 4.3	68.8 ~ 90.2	5.5	75.5 ± 3.1	68.8 ~ 85.5	4.1	4.0**
		**			ns			**			

** and * indicate significance at *P* = 0.01 and *P* = 0.05, respectively; *ns* not significant, *CV* Coefficient of variation

The phenotypic pair-wise correlations between each pair of the 11 ECQ traits in three panels and 2 years are given in Additional file 1: Table S2. The correlations of the same trait were almost all significant in three panels between both years indicating environmental stability of these traits. The correlation coefficients were generally larger in the whole population and *indica* subpopulation than in the *japonica* subpopulation. In both year, except for ASV, AC was negatively correlated with GC, PV and BDV and was positively correlated with HPV, CPV, SBV, CSV, PeT and Ptemp in more than two panels. GC had significant correlations with most of RVA parameters except for PV and ASV in both years. Significant correlations for ASV were mainly identified with PV, BDV, PeT and Ptemp in whole population and the *japonica* subpopulation in both years. Each pair of most of RVA parameters showed significant correlations in more than two panels, but there were no or weak correlations for PV with CSV and PeT in three panels in both years.

### Association analysis

The strong population structure between the two subspecies of cultivated rice makes it inappropriate to look for association loci in the entire population. We thus conducted association analysis separately for *indica* and *japonica* subpopulations based on the Q + K mix linear model.

For *indica* subpopulation, 33 associated loci influencing nine traits were totally identified with *P* < 2.4 × 10^− 4^, of which 16 loci were detected in both years (Table 2). There were seven strong association signals with *P* < 10^− 7^. For AC and GC, the same four loci were identified on the short arm of Chr.6 which were markers of *Wx* gene or tightly linked with *Wx* gene. Four loci for AC were detected in both years with the phenotypic variance explained of 7.67 to 17.24%. In four loci for GC, only *RM588* was found in both years explaining 6.85 and 9.12% of the phenotypic variance respectively, while the other three loci were detected only in 2016 with a minor phenotypic variance explained of 2.71 to 4.97%. Two markers (*SSIIa-S1* and *SSIIa-S2*) from the *OsSSIIa* gene affecting ASV were found, explaining the phenotypic variance of approximately 17.0% in 2015. Eighteen loci for five viscosity parameters (PV, BDV, CPV, SBV and CSV) were identified across 2 years of which 10 were detected in both years, with phenotypic variation explained by each locus varying from 4.09 to 10.45%. Five loci for PeT were detected of which four were found in 2015 and one in 2016, explaining the phenotypic variance of 7.96 to 17.82%.

**Table 2:** Determination of loci for eating and cooking qualities in *indica* subpopulation

Traits	Associated Markers	Position (kb)	Chr.	MAF	2015		2016		Linked Gene
					*P* value	*R*^*2*^ (%)	*P* value	*R*^*2*^ (%)	
AC	RM170	1361	6	0.099	4.47E-06	7.67	6.74E-07	7.93	*Wx* linked
	RM588	1612	6	0.025	3.29E-18	17.24	7.85E-18	15.83	*Wx* linked
	Wx-S1	1765	6	0.346	3.53E-11	10.93	3.03E-10	9.4	*Wx*^*a*^*/Wx*^*b*^
	Wx-S2	1765	6	0.012	1.35E-06	9.45	2.97E-06	8.43	(CT)_n_ in *Wx*
GC	RM170	1361	6	0.099			1.83E-05	4.97	*Wx* linked
	RM588	1612	6	0.025	7.77E-06	7.92	2.06E-08	7.15	*Wx* linked
	Wx-S1	1765	6	0.346			3.84E-04	2.71	*Wx*^*a*^*/Wx*^*b*^
	Wx-S2	1765	6	0.012			7.30E-04	4.43	(CT)_n_ in *Wx*
ASV	SSIIa-S1	6747	6	0.457	3.10E-10	17.0	2.30E-04	7.83	*SSIIa*
	SSIIa-S2	6747	6	0.012	2.71E-09	17.04			*SSIIa*
PV	RM588	1612	6	0.025	3.91E-04	8.48			*Wx* linked
	Wx-S2	1765	6	0.012	9.84E-04	8.97	5.84E-04	9.89	(CT)_n_ in *Wx*
	RM412	30,328	6	0.438			3.51E-04	6.07	*SBE1* linked
	RM346	21,579	7	0.012			4.74E-04	8.22	
BDV	RM170	1361	6	0.099	5.13E-04	5.72	7.45E-04	5.39	*Wx* linked
	RM588	1612	6	0.025	8.79E-08	10.45	2.41E-05	7.41	*Wx* linked
	Wx-S1	1765	6	0.346	8.81E-05	4.99			*Wx*^*a*^*/Wx*^*b*^
CPV	RM170	1361	6	0.099	4.18E-06	7.62	7.38E-05	5.98	*Wx* linked
SBV	RM170	1361	6	0.099	1.91E-06	7.26	7.92E-06	6.55	*Wx* linked
	RM588	1612	6	0.025	4.42E-10	10.45	3.24E-08	8.96	*Wx* linked
	Wx-S1	1765	6	0.346	5.18E-06	5.37	3.26E-05	4.49	*Wx*^*a*^*/Wx*^*b*^
	Wx-S2	1765	6	0.012	9.26E-04	5.48			(CT)_n_ in *Wx*
	RM225	3417	6	0.013			6.40E-04	4.6	*SS1* linked
CSV	RM170	1361	6	0.099	4.73E-06	8.23	8.80E-04	6.24	*Wx* linked
	RM588	1612	6	0.025	4.57E-06	8.38	1.08E-05	9.42	*Wx* linked
	Wx-S1	1765	6	0.346	2.47E-04	4.37	4.10E-04	4.78	*Wx*^*a*^*/Wx*^*b*^
	RM3262	22,469	8	0.012	3.92E-04	4.09			
	RM216	5336	10	0.069	7.89E-04	5.27			
PeT	RM488	24,808	1	0.013	1.06E-05	12.93			
	RM5473	31,676	4	0.075	5.11E-06	14.29			
	SSI-S1	3080	6	0.431	1.19E-08	17.82			*SSI*
	SSIIa-S2	6747	6	0.012			8.14E-04	7.96	*SSIIa*
	RM189	22,095	9	0.073	3.04E-05	11.8			

*MAF* Minor allele frequency

*R*^*2*^ represents the proportion of phenotypic variation explained by each associated locus

For *japonica* subpopulation, a total of 30 markers were identified as the association signals with *P* < 2.4 × 10^− 4^, of which 8 loci were detected in both years (Table 3). For AC, two loci (*Wx-S3* and *RM550*) were detected in both years and the *Wx-S3* loci had the largest effect with the phenotypic variance explained of 21.86 and 20.87% in 2015 and 2016, respectively. Four loci for ASV were detected only in 2015 contributing minor effects with the phenotypic variance explained of 3.55 to 5.56%, of which two markers were derived from the *OsSSIIa* gene. Sixteen loci for six viscosity parameters (PV, HPV, BDV, CPV, SBV and CSV) were totally identified across 2 years. Only the *Wx-S3* locus controlling HPV, CPV, SBV and CSV was detected in both years, while other loci were found in 1 year. The detected loci in each viscosity parameter varied from two for BDV, CPV and CSV to four for HPV. For PeT, three loci were identified, of which only the *Wx-S3* locus was found in both years. Five loci for Ptemp were detected across 2 years. The locus *SSIIIb-S1* was identified only in 2015 which explained 7.51% of the phenotypic variance. The locus *RM276* found in both years explained 6.85 and 9.12% of the phenotypic variance in 2015 and 2016, respectively. Three additional loci (*SSIIa-S1*, *SSIIa-S2* and *RM5688*) were detected only in 2016 accounted for 3.48, 6.17 and 4.52% of the phenotypic variance, respectively.

**Table 3:** Determination of loci for eating and cooking qualities in *japonica* subpopulation

Traits	Associated Markers	Position (kb)	Chr.	MAF	2015		2016		Linked Gene
					*P* value	*R*^*2*^	*P* value	*R*^*2*^	
AC	Wx-S3	1765	6	0.058	1.81E-25	21.86	3.12E-28	20.87	*Wx*^*mp*^
	RM550	12,464	2	0.353	9.36E-06	5.14	1.40E-05	4.39	
ASV	SSIIa-S1	6747	6	0.035	3.84E-04	3.55			*SSIIa*
	SSIIa-S2	6747	6	0.029	4.27E-05	5.56			*SSIIa*
	RM5331	7177	6	0.052	3.54E-04	3.6			*SSIIa* linked
	RM1375	16,716	10	0.012	9.23E-04	5.39			*SSIIb* linked
PV	RM302	32,988	1	0.006			7.44E-04	3.54	
	RM5791	10,747	2	0.012			5.27E-04	2.59	
	RM420	30,092	7	0.017			9.88E-04	2.92	
HPV	SBE4-S2	20,238	4	0.012	4.56E-05	5.04			*SBE4*
	AGPLar	28,877	5	0.006	3.84E-04	4.87			*AGPLar*
	Wx-S3	1765	6	0.058	3.19E-04	3.97	9.00E-04	2.58	*Wx*^*mp*^
	STS9–1	10,861	9	0.012	3.28E-04	3.96			
BDV	Wx-S3	1765	6	0.058			8.16E-05	4.43	*Wx*^*mp*^
	RM276	6230	6	0.012			8.38E-04	4.79	*SSIIa* linked
CPV	SBE4-S2	20,238	4	0.012	2.50E-04	4.34			*SBE4*
	Wx-S3	1765	6	0.058	1.22E-10	12.05	3.17E-07	6.34	*Wx*^*mp*^
SBV	RM550	12,464	2	0.353	8.93E-05	4.46			
	Wx-S3	1765	6	0.058	4.53E-08	8.11	7.74E-10	9.37	*Wx*^*mp*^
	RM336	21,872	7	0.018			3.67E-04	6.05	
CSV	RM550	12,464	2	0.353	3.49E-04	4.03			
	Wx-S3	1765	6	0.058	5.20E-14	15.48	1.71E-11	12.36	*Wx*^*mp*^
PeT	SBE4-S2	20,238	4	0.012	8.65E-04	3.45			*SBE4*
	Wx-S3	1765	6	0.058	4.78E-06	6.32	2.93E-05	4.73	*Wx*^*mp*^
	RM276	6230	6	0.012			2.79E-05	6.42	*SSIIa* linked
Ptemp	SSIIIb-S1	31,759	4	0.017	1.82E-06	7.51			*SSIIIb*
	RM276	6230	6	0.012	2.73E-05	6.85	7.12E-10	9.12	*SSIIa* linked
	SSIIa-S1	6747	6	0.035			6.06E-05	3.48	*SSIIa*
	SSIIa-S2	6747	6	0.029			3.68E-07	6.17	*SSIIa*
	RM5688	1716	9	0.006			9.46E-05	4.52	

MAF: Minor allele frequency

*R*^*2*^ represents the proportion of phenotypic variation explained by each associated locus

**Fig. 3:**
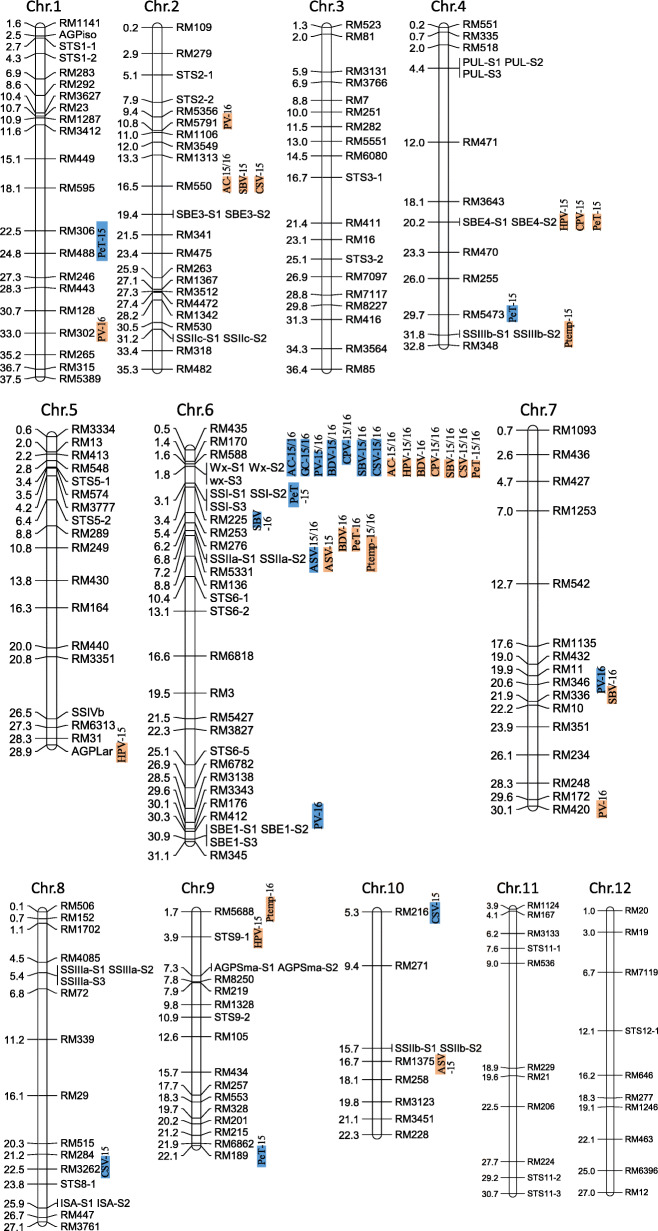
The distribution of association loci for eating and cooking qualities in 12 chromosomes of rice. The blue and brown boxes indicated the loci detected in *indica* and *japonica* subpopulations, respectively

Some association signals for the investigated traits were located nearby the known SSRGs (*Wx*, *SSIIa*, *SSI*, *SBE1*, *SSIIb*), for example *RM170*, *RM588*, *RM225*, *RM276*, *RM412* and *RM1375*. Figure 3 exhibits the location of these association signals on rice chromosomes separately from the *indica* and *japonica* subpopulations. The most association signals derived from two subpopulations across 2 years were assembled in *Wx* gene. Notably, we regarded those loci that were detected with the physical distance less than 2.0 Mb as the same QTL, and these loci in different subpopulations were regarded as alleles.

## Discussion

There is an ancient and well-established divergence between two major subspecies of *Oryza sativa*, *indica* and *japonica* [22]. Chen et al. [23] developed a set of SNPs and uncovered the well-known subspecific differentiation and geographic differentiations within *indica* and *japonica* germplasms. For rice eating quality characteristics, a wide range of varietal differences have been recognized in worldwide rice germplasms including *indica* and *japonica* rice cultivars [24]. These rice germplasm provide abundant gene resources for the discovery of genes that control important traits.

In the present study, a set of 253 non-glutinous germplasm of rice cultivars including 83 *indica* and 170 *japonica* accessions were investigated for allelic differences of SSRGs and phenotypic variation of ECQs. The *indica* cultivars contain both homozygous alleles of *Wx*^*a*^ (in a large amount of accessions) and *Wx*^*b*^ (in a small amount of accessions) with a wide range of AC from 12.5 to 28.5%, while all the *japonica* cultivars have the homozygous *Wx*^*b*^ allele with AC ranging from 8.0 to 19.0%. This indicates that a large variation in AC existed with interspecies and intraspecies of rice cultivars and AC would be controlled by genetic factors other than *Wx* locus [25, 26]. Genome-wide association mapping coupled with candidate genes was utilized for identifying new genetic factors and verifying effects of the known genes on investigated traits. Given that the linkage disequilibrium could be caused by the admixture of subpopulations and might result in a high number of false positives [27], population structure has to be estimated in the association analysis. Cultivated rice has undergone a long-term domestication and evolution, artificial selection and different ecological conditions contributed to the differentiation of cultivated rice, especially the subspecific differentiation [28, 29]. As expected, a strong population differentiation was detected between two subspecies of *indica* and *japonica* rice cultivars in this study. For the *japonica* subpopulation, two *indica* accessions from South Korea that harbored *Wx*^*b*^ allele were grouped with all the *japonica* accessions, indicating the similar genetic background with *japonica* rice. The different loci or alleles associated with the same ECQ trait in the two subpopulations provided access to compare genetic factors between *indica* and *japonica* background.

Similar to previous reports, the *Wx* gene was responsible for most of the ECQ traits including AC, GC, PV, HPV, BDV, CPV, SBV, CSV and PeT either in *indica* or *japonica* subpopulation [15]. For AC, *Wx-S1* (*Wx*^*a*^/*Wx*^*b*^ allele) and *Wx-S2* (CT variation) were not the most significant associated sites identified in *indica* subpopulation, but *RM588*, the *Wx* adjacent marker, was the major locus explaining the largest phenotypic variation. *RM170* was the most upstream associated locus for AC identified in *indica* subpopulation and was recognized as a *Wx* allele because of their close positions, suggesting that the difference of the upstream regulatory region of *Wx* gene could partly explain AC variation among *indica* rice accessions. In contrast, two association loci (*Wx-S3* and *RM550*) for AC detected in *japonica* subpopulation were different with those detected in *indica* subpopulation. The *Wx-S3* loci was mapped for AC in *japonica* subpopulation for both year. *Wx*^*mp*^ was an allele mutated from G to A in exon 4 of *Wx*^*b*^ resulting in the dramatically decrease of amylose content [30]. Due to lower amylose content, the carrying rice materials presented higher stickiness and lower hardness than the rice varieties with *Wx*^*b*^. *RM550* on rice chromosome 2 was the other associated loci for AC identified in *japonica* subpopulation across both years. A QTL, *qAC2*, flanked by *RM3730* and *RM6933* on rice chromosome 2 has been previously detected and fine-mapped in populations derived from crosses between Koshihikari and Akihikari [26, 31]. By comparing their genomic region, *RM550* and *qAC2* were not the same QTL. The allelic differences of *Wx-S3* and *RM550* could give the main explanation of AC variation among the *japonica* rice accessions.

Four associated loci for GC were detected in *indica* subpopulation and *RM588* was the locus explaining the largest phenotypic variation of GC, which were the same loci as identified for AC in *indica* subpopulation. This was in agreement with previous findings that GC was mainly controlled by the *Wx* alleles [11]. But no locus for GC was detected in *japonica* subpopulation, probably because of lower phenotypic variation among these rice accessions.

For ASV, two association loci *SSIIa-S1* and *SSIIa-S2* detected in *indica* subpopulation showed a high proportion of phenotypic variation, while four loci *SSIIa-S1*, *SSIIa-S2*, *RM5331* and *RM1375* identified in *japonica* population had a minor proportion of phenotypic variation. The loci *RM5331* and *RM1375* were linked with the soluble starch syntheses genes *SSIIa* and *SSIIb* respectively. This result indicated that *SSIIa* alleles were randomly distributed in both *indica* and *japonica* subpopulations, even though the ASV average in *japonica* subpopulation was higher than that in *indica* subpopulation. From the previous reports, allelic variation in *SSIIa* was identified by exploring the interspecies genetic variation between *indica* and *japonica*, due to the intermediate length enrichment of amylopectin chains [2, 3]. In the present study, allelic variations of *SSIIa* without the tendency of subspecies might be due to gene introgression between *indica* and *japonica* varieties during long-term domestication and artificial selection [32].

Most of loci for RVA viscosity parameters detected in both subpopulations were SSRGs or adjacent with SSRGs. Of these, *Wx* alleles and its neighboring markers were mapped by most of RVA traits from both subpopulations, which was consistent with previous reports [33–36]. Other loci were separately located on different chromosome regions. For example, the loci for BDV, PeT and Ptemp associated with *SSIIa*, the loci for HPV, CPV and PeT associated with *SBE4*, the locus for Ptemp associated with *SSIIIb* and the locus for HPV associated with *AGPLar* were found in *japonica* subpopulation. The loci for SBV and PeT associated with *SSI* and the locus for PV associated with *SBE1* were identified in *indica* subpopulation. Notably, in addition to AC, *RM550* was also associated with SBV and CSV in *japonica* subpopulation, suggesting that it was an important locus for improving eating qualities in rice materials with *japonica* background. Other loci for RVA parameters associated with SSR markers were detected in individual subpopulation and only in 1 year, which distributed on Chr.1, 4, 7, 8, 9 and 10. The accuracy of these loci needs to be verified in future, even though most of them have been identified in natural or linkage populations based on different genetic background [15, 37–39].

## Conclusions

We described a strategy to search for specific genetic factors of rice ECQs in rice subpopulations of *indica* and *japonica* cultivars. *Wx* alleles had the largest effect on AC, GC and most pasting properties. The novel associated locus *RM550* on chromosome 2 identified in *japonica* subpopulation displayed the minor effects on rice AC and RVA pasting, which was irrespective of the effect of *Wx*. The associated alleles of SSRGs or their adjacent markers, *RM588*, *Wx-(CT)*_*n*_, *SSI* and *SBE1* for *indica* subpopulation and *RM550*, *Wx*^*mp*^, *SSIIa* and *SBE4* for *japonica*, were specific genetic factors for rice subspecies, which can provide efficiency and precision in marker-assisted selection for *indica* and *japonica* breeding. The availability of these markers will facilitate the development of new rice varieties with the desired ECQ traits.

## Methods

### Plant materials and field planting

A total of 253 non-glutinous cultivated rice varieties, comprising 83 *indica* and 170 *japonica* accessions were used for the present study. Their country wise accessions are as follows; 21 from Japan, 9 from South Korea, 7 from Philippines, 4 from Cambodia, 4 from India, 4 from Indonesia, 3 from Bangladesh, 2 from Vietnam, 2 from Myanmar and 197 from P. R. China. All rice accessions were planted in 2015 and 2016 during rice growing season from May to November at Jiangsu Academy of Agricultural Sciences farm (32^o^1’49″N, 118^o^52’24″E), China. Each accession was planted in 4 rows with 12 plants per row. A randomized block design with two replications for each cultivar was followed.

After being air-dried and stored at room temperature for 2.5 months, all rice samples were dehusked to brown rice with a rice huller (SY88-TH, Sanyong, South Korea) and subsequently polished using a grain polisher (Kett, Tokyo, Japan). The polished rice was ground to powder in a mill (Foss 1093 Cyclotec Sample Mill, Sweden) and sieved through a 100-mesh sieve to obtain rice flour samples for phenotypic measurement.

### Phenotypic evaluation

The AC was determined using the iodine staining method described in the European Standard EN ISO 6647-2-2015. The AC was calculated using a standard curve made from four rice samples with known AC. ASV was measured visually by soaking the milled rice grains in 1.7% KOH for 23 h at 30 °C [40]. The GC was determined following the method of Cagampang et al. [41] with minor modifications. RVA profile was determined using a Cereal Visco Analyzer (TechMaster, Perten, Sweden). The heating profile was setup according to the method of AACC61–01 and 61–02. The output RVA parameters include peak viscosity (PV), hot paste viscosity (HPV), cool paste viscosity (CPV), pasting temperature (Ptemp) and peak time (PeT). Three secondary parameters were calculated from the output parameters: breakdown viscosity (BDV = PV - HPV), setback viscosity (SBV = CPV - PV) and consistency viscosity (CSV = CPV - HPV). Each trait was measured in three replicates.

### Molecular marker genotyping

Young leaves at the tillering stage were collected from one seedling of each cultivar and genomic DNA was extracted with the CTAB extraction buffer. For genotyping, a total of 210 molecular markers, comprising 157 SSR (simple sequence repeat), 19 STS (sequence tagged site) and 34 genic markers derived from 15 SSRGs were used (Additional file 1: Table S3).

### Genetic diversity, phylogenetic analysis and population structure

The summary statistics including the number of alleles per locus, major allele frequency, gene diversity, PIC values, and classical *F*st values were determined using Power Marker version 3.25 [42]. Nei’s distance [43] was calculated and used for the unrooted phylogeny reconstruction using neighbor-joining method as implemented in Power Marker with the tree viewed using MEGA 4.0 [44].

Analysis of population structure among rice accessions was performed using the software package STRUCTURE V2.2 [45, 46]. The optimum number of populations (K) were selected after five independent runs of a burn-in length of 50,000 followed by 10,000 iterations for each value of K (testing from K = 2 to K = 10). A model-based clustering method was applied for identifying subgroups with distinctive allele frequencies and setting each individual into different K cluster, where K is chosen in advance but can be varied for independent runs of the algorithm. The most likely number of cluster (K) was selected by comparing with the logarithmized probabilities of data (Pr [X|K]) [47].

### Association analysis

To compare phenotypes of two subpopulations identified by STRUCTURE V2.1, ANOVA was employed with the SAS program version 8 (SAS Institute Inc., Cary, NC), followed by multiple means comparisons. To control for false positives, the Q + K mixed linear model was used for the genome-wide association mapping with TASSEL V5.0 [48]. The Q matrix was obtained from the analysis results of STRUCTURE V2.1. The K matrix was generated from the analysis results using the kinship matrix function in TASSEL V5.0. The *P* value determines whether a marker (QTL) is significantly associated with the trait and the –Log*P* value higher than 4 was set as a threshold for strong associations. The *R*^*2*^ (the fraction of the total variation explained by the marker) for each locus was estimated using TASSEL 5.0.

## Supplementary information

**Additional file 1: Table S1.** The allele number, gene diversity and polymorphism information content of each molecular marker genotyping in 253 accessions. **Table S2.** Pairwise correlation values among 11 eating and cooking quality traits in the whole population and two subpopulations in 2015 and 2016. **Table S3.** The polymorphic molecular markers and sequences for tagging starch synthesis-related genes.

**Additional file 2: Figure S1.** The pattern of polymorphism shown by the molecular markers in rice population. **Figure S2.** The analysis of linkage disequilibrium (LD) patterns among the *indica* and *japonica* subpopulations genotyping with 210 molecular markers.

## Data Availability

All the rice materials generated and/or analysed during the current study are available in the Jiangsu Provincial Platform for Conservation and Utilization of Agricultural Germplasm (http://jagis.jaas.ac.cn/). The raw phenotype data and genotype data are included in this manuscript and its additional files.

## Abbreviations

ECQ: Eating and cooking quality
AC: Amylose content;
GC: Gel consistency
GT: Gelatinization temperature
RVA: Rapid Visco Analyzer
SSRG: Starch synthesis related gene
QTL: Quantitative trait loci
PV: Peak viscosity
HPV: Hot paste viscosity
CPV: Cool paste viscosity
Ptemp: Pasting temperature
PeT: Peak time
BDV: Breakdown viscosity
SBV: Setback viscosity
CSV: Consistency viscosity
ASV: Alkali spreading value
CV: Coefficient of variation
PIC: Polymorphism information content
SSR: Simple sequence repeat
STS: Sequence tagged site

## References

[1] Bao J (2012). Toward understanding the genetic and molecular bases of the eating and cooking qualities of rice. Cereal Foods World.

[2] Umemoto T, Aoki N, Lin H, Nakamura Y, Inouchi N, Sato Y (2004). Natural variation in rice *starch synthase IIa* affects enzyme and starch properties. Funct Plant Biol.

[3] Umemoto T, Yano M, Satoh H, Shomura A, Nakamura Y (2002). Mapping of a gene responsible for the difference in amylopectin structure between japonica-type and indica-type rice varieties. Theor Appl Genet.

[4] Hiroyuki S, Yasuhiro S, Makoto S, Tokio I (2002). Molecular characterization of *Wx-mq*, a novel mutant gene for low-amylose content in endosperm of rice (*Oryza sativa* L.). Breed Sci.

[5] Mikami I, Uwatoko N, Ikeda Y, Yamaguchi J, Hirano HY, Suzuki Y (2008). Allelic diversification at the *wx* locus in landraces of Asian rice. Theor Appl Genet.

[6] Liu L, Ma X, Liu S, Zhu C, Jiang L, Wang Y (2009). Identification and characterization of a novel waxy allele from a Yunnan rice landrace. Plant Mol Biol.

[7] Ayres NM, Mcclung AM, Larkin PD, Bligh HFJ, Jones CA, Park WD (1997). Microsatellites and a single-nucleotide polymorphism differentiate apparent amylose classes in an extended pedigree of us rice germ plasm. Theor Appl Genet.

[8] Isshiki M, Nakajima M, Satoh H, Shimamoto K (2000). Dull: Rice mutants with tissue-specific effects on the splicing of the waxy pre-mRNA. Plant J.

[9] Isshiki M, Matsuda Y, Takasaki A, Wong HL, Satoh H, Shimamoto K (2008). *Du3*, a mRNA cap-binding protein gene, regulates amylose content in japonica rice seeds. Plant Biotech.

[10] Cuevas RP, Fitzgerald MA. Genetic diversity of rice grain quality, genetic diversity in plants. IntechOpen. 2012:286–310. 10.5772/35119.

[11] Su Y, Rao Y, Hu S, Yang Y, Gao Z, Zhang G (2011). Map-based cloning proves qGC-6, a major QTL for gel consistency of japonica/indica cross, responds by waxy in rice (*Oryza sativa* L.). Theor Appl Genet.

[12] Wang L, Liu W, Xu Y, He Y, Luo L, Xing Y (2007). Genetic basis of 17 traits and viscosity parameters characterizing the eating and cooking quality of rice grain. Theor Appl Genet.

[13] Tian Z, Qian Q, Liu Q, Yan M, Liu X, Yan C (2009). Allelic diversities in rice starch biosynthesis lead to a diverse array of rice eating and cooking qualities. Proc Natl Acad Sci U S A.

[14] Yan B, Tondi Yacouba N, Chen J, Wang Y, Gao G, Zhang Q (2014). Analysis of minor quantitative trait loci for eating and cooking quality traits in rice using a recombinant inbred line population derived from two *indica* cultivars with similar amylose content. Mol Breed.

[15] Xu F, Sun C, Huang Y, Chen Y, Tong C, Bao J (2015). QTL mapping for rice grain quality: a strategy to detect more QTLs within sub-populations. Mol Breed.

[16] Gao Z, Zeng D, Cheng F, Tian Z, Guo L, Su Y (2011). *ALK*, the key gene for gelatinization temperature, is a modifier gene for gel consistency in rice. J Integr Plant Biol.

[17] Bao J, Corke H, Sun M (2006). Nucleotide diversity in starch synthase IIa and validation of single nucleotide polymorphisms in relation to starch gelatinization temperature and other physicochemical properties in rice (*Oryza sativa* L.). Theor Appl Genet.

[18] Han Y, Xu M, Liu X, Yan C, Korban S, Chen X (2004). Genes coding for starch branching enzymes are major contributors to starch viscosity characteristics in waxy rice (*Oryza sativa* L.). Plant Sci.

[19] Yan C, Tian Z, Fang Y, Yang Y, Li J, Zeng S (2011). Genetic analysis of starch paste viscosity parameters in glutinous rice (*Oryza sativa* L.). Theor Appl Genet.

[20] Yao S, Zhang Y, Liu Y, Zhao C, Zhou L, Chen T (2020). Allelic effects on eating and cooking quality of *SSIIa* and *PUL* genes under *Wx*^*mp*^ background in rice. Chin J Rice Sci.

[21] Wang H, Xu X, Vieira FG, Xiao Y, Li Z, Wang J (2016). The power of inbreeding: NGS-based GWAS of rice reveals convergent evolution during rice domestication. Mol Plant.

[22] Huang X, Wei X, Sang T, Zhao Q, Feng Q, Zhao Y (2010). Genome-wide association studies of 14 agronomic traits in rice landraces. Nat Genet.

[23] Chen H, He H, Zou Y, Chen W, Yu R, Liu X (2011). Development and application of a set of breeder-friendly SNP markers for genetic analyses and molecular breeding of rice (*Oryza sativa* L.). Theor Appl Genet.

[24] Nakamura S, Suzuki D, Kitadume R, Ohtsubo K (2012). Quality evaluation of rice crackers based on physicochemical measurements. Biosci Biotechnol Biochem.

[25] Ando I, Sato H, Aoki N, Suzuki Y, Hirabayashi H, Kuroki M (2010). Genetic analysis of the low­amylose characteristics of rice cultivars Oborozuki and Hokkai­PL9. Breed Sci.

[26] Takemoto-Kuno Y, Mitsueda H, Suzuki K, Hirabayashi H, Ideta O, Aoki N (2015). *qAC2*, a novel QTL that interacts with *Wx* and controls the low amylose content in rice (*Oryza sativa* L.). Theor Appl Genet.

[27] Yu J, Pressoir G, Briggs WH, Bi IV, Yamasaki M, Doebley JF (2006). A unified mixed-model method for association mapping that accounts for multiple levels of relatedness. Nat Genet.

[28] Wang J, Wan X, Li H, Pfeiffer W, Crouch J, Wan J (2007). Application of identified QTL-marker associations in rice quality improvement through a design-breeding approach. Theor Appl Genet.

[29] Huang X, Kurata N, Wei X, Wang Z, Wang A, Zhao Q (2012). A map of rice genome variation reveals the origin of cultivated rice. Nature..

[30] Yang J, Wang J, Fan F, Zhu J, Chen T, Wang C (2013). Development of AS-PCR marker based on a key mutation confirmed by resequencing of *Wx-mp* in milky princess and its application in *japonica* soft rice (*Oryza sativa* L.) breeding. Plant Breed.

[31] Kobayashi A, Tomita K, Yu F, Takeuchi Y, Yano M (2008). Verification of quantitative trait locus for stickiness of cooked rice and amylose content by developing near­isogenic lines. Breed Sci.

[32] Zhao K, Wright M, Kimball J, Eizenga G, McClung A, Kovach M (2010). Genomic diversity and introgression in *O. sativa* reveal the impact of domestication and breeding on the rice genome. PLoS One.

[33] Bao J, Zheng X, Xia Y, He P, Shu Q, Lu Y (2000). QTL mapping for the paste viscosity characteristics in rice (*Oryza sativa* L.). Theor Appl Genet.

[34] Larkin PD, McClung AM, Ayres NM, Park WD (2003). The effect of the waxy locus (granule bound starch synthase) on pasting curve characteristics in specialty Rices (*Oryza sativa* L.). Euphytica..

[35] Chen M, Bergman CJ, Pinson SRM, Fjellstrom RG (2008). Waxy gene haplotypes: associations with pasting properties in an international rice germplasm collection. J Cereal Sci.

[36] Traore K, Mcclung AM, Chen MH, Fjellstrom R (2011). Inheritance of flour paste viscosity is associated with a rice waxy gene exon 10 SNP marker. J Cereal Sci.

[37] Yao X, Wang J, Liu J, Zhang J, Ren C, Ma D (2017). Mapping quantitative trait loci associated with starch paste viscosity in rice (*Oryza sativa* L.) under different environmental conditions. Plant Breed.

[38] Xu F, Bao J, He Q, Park YJ (2016). Genome-wide association study of eating and cooking qualities in different subpopulations of rice (*Oryza sativa* L.). BMC Genomics.

[39] Wang H, Zhu S, Dang X, Liu E, Hu X, Eltahawy MS (2019). Favorable alleles mining for gelatinization temperature, gel consistency and amylose content in *Oryza sativa* by association mapping. BMC Genet.

[40] Little RR, Hilder GB, Daw Son EH (1958). Differential effect of dilute alkali on 25 varieties of milled white rice. Cereal Chem.

[41] Cagampang GB, Perez CM, Juliano BO (1973). A gel consistency test for eating quality of rice. J Sci Food Agric.

[42] Liu K, Muse SV (2005). PowerMarker: an integrated analysis environment for genetic marker analysis. Bioinformatics..

[43] Nei M, Tajima F, Tateno Y (1983). Accuracy of estimated phylogenetic trees from molecular data II. Gene frequency data. J Mol Evol.

[44] Tamura K, Dudley J, Nei M, Kumar S (2007). MEGA4: molecular evolutionary genetics analysis (MEGA) software version 4.0. Mol Biol Evol.

[45] Pritchard JK, Wen W (2004). Documentation for structure software: version 2.

[46] Falush D, Stephens M, Pritchard JK (2003). Inference of population structure using multilocus genotype data: linked loci and correlated allele frequencies. Genetics..

[47] Pritchard JK, Stephens M, Donnelly P (2000). Inference of population structure using multilocus genotype data. Genetics..

[48] Bradbury PJ, Zhang Z, Kroon DE, Casstevens TM, Ramdoss Y, Buckler ES (2007). TASSEL: software for association mapping of complex traits in diverse samples. Bioinformatics..

