# Supplementary material for: Genetic dissection of eating and cooking qualities in different subpopulations of cultivated rice (Oryza sativa L.) through association mapping
Source: BMC Genet. 2020 Oct 14;21:119. doi: 10.1186/s12863-020-00922-7 (PMC7556922; doi:10.1186/s12863-020-00922-7)
Supplement: Supplementary file 2 — Additional file 2: Figure S1. The pattern of polymorphism shown by the molecular markers in rice population. Figure S2. The analysis of linkage disequilibrium (LD) patterns among the indica and japonica subpopulations genotyping with 210 molecular markers. [file 12863_2020_922_MOESM2_ESM.docx]

**Fig. S1** The pattern of polymorphism shown by the molecular markers in rice whole population

**Fig. S2** The analysis of linkage disequilibrium (LD) patterns among the *indica* subpopulation (a) and *japonica* subpopulation (b). The section above the diagonal indicates the standardized disequilibrium coefficients (*R^2^*); while the section below the diagonal indicates the *P* value size of the testing LD as shown in the color code at the lower right: white *P*>0.05, blue 0.05>*P*>0.01, green 0.01>*P*>0.001 and red *P* <0.001.
